# Conditional trust: Community perceptions of drone use in malaria control in Zanzibar

**DOI:** 10.1016/j.techsoc.2022.101895

**Published:** 2022-02

**Authors:** Andy Hardy, Mark Proctor, Cathryn MacCallum, Josh Shawe, Safia Abdalla, Rajab Ali, Salha Abdalla, Gregory Oakes, Laura Rosu, Eve Worrall

**Affiliations:** a Department of Geography and Earth Sciences, Aberystwyth University, UK; b Sazani Consulting, Camarthen, UK; c Sazani Trust, Zanzibar, United Republic of Tanzania; d Liverpool School of Tropical Medicine, Liverpool, UK

**Keywords:** Drones, Unmanned aerial vehicles, Malaria, Community perceptions, Public health, Vector control

## Abstract

**Background:**

The potential of drones to support public health interventions, such as malaria vector control, is beginning to be realised. Although permissions from civil aviation authorities are often needed for drone operations, the communities over which they fly tend to be ignored: How do affected communities perceive drones? Is drone deployment accepted by communities? How should communities be engaged?

**Methods:**

An initiative in Zanzibar, United Republic of Tanzania is using drones to map malarial mosqutio breeding sites for targeting larval source management interventions. A community engagement framework was developed, based on participatory research, across three communities where drones will be deployed, to map local perceptions of drone use. Costs associated with this exercise were collated.

**Results:**

A total of 778 participants took part in the study spanning a range of community and stakeholder groups. Overall there was a high level of acceptance and trust in drone use for public health research purposes. Despite this level of trust for drone operations this support was conditional: There was a strong desire for pre-deployment information across all stakeholder groups and regular updates of this information to be given about drone activities, as well as consent from community level governance. The cost of the perception study and resulting engagement strategy was US$24,411.

**Conclusions:**

Mapping and responding to community perceptions should be a pre-requisite for drone activity in all public health applications and requires funding. The findings made in this study were used to design a community engagement plan providing a simple but effective means of building and maintaining trust and acceptability. We recommend this an essential investment.

## Highlights

• There are a lack of studies into the community perceptions of drone use in a rural sub-Saharan setting.
• There is widespread trust in Zanzibar for the use of drones in public health but this trust comes with certain conditions.
• Communities need to be informed of drone activities and consent granted at the community level.
• Trust and consent for drone operations can be easily undone, especially where the use of this technology is unsolicited.
• Mapping community perceptions and establishing an engagement plan should be a pre-requisite for all drone operations.

## Graphical abstract

**Figure:**
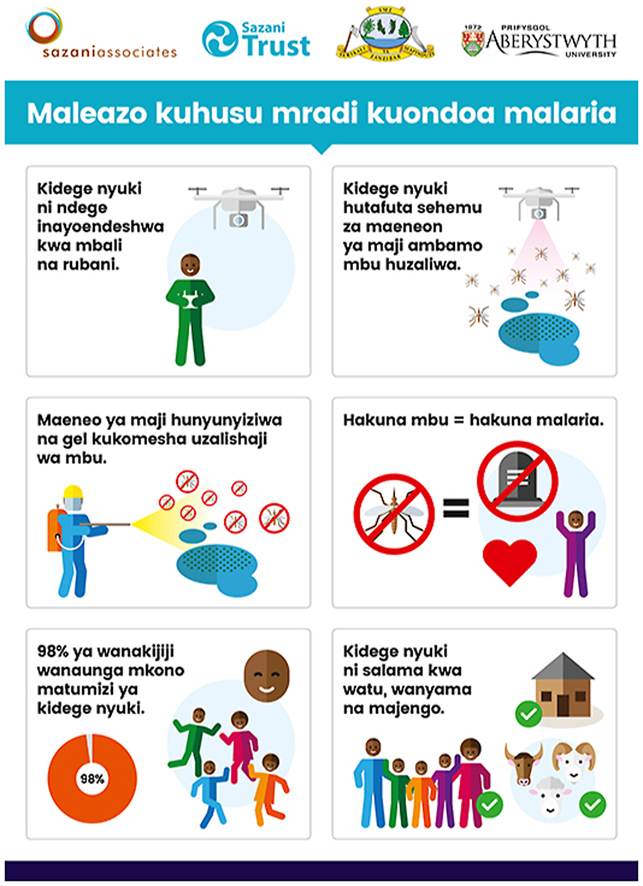

## 1 Introduction

Drone technology has the potential to provide benefits for a wide range of sectors and applications. The advantages that drones can offer to the public health sector have been acknowledged and their integration into practical public health interventions and operations are beginning to be realised, notably in the way medical supplies can be delivered and how disease risk can be mapped and controlled [[1], [2], [3], [4], [5], [6], [7]]. In malaria control, there is growing evidence that drones can be used for mapping mosquito vector habitats [[1], [2], [3], [4],^7^] potentially providing a step-change in the way that vector control interventions are delivered.

Ethically there are major considerations for utilizing drones, particularly in countries where they have the potential to be used for cohersive or warfare purposes or where they do not represent indigenoius or accessible technology, adding to the apparent power differential of technological equipment (including drones) deployed from the global North into communities in the global South [8]. From a public perspective, one of the main sources of mistrust relate to the misuse of drones for invading privacy, espciecially related to their use in commercial or hobby applications, together with their potential misuse by criminals and terrorists [[9], [10], [11]]. Interestingly, these negative connotations are potentially augmented by actual public knowledge being significantly less than perceived knowledge [9].

A series of ethical guidelines were issued in 2016, by the Council for International Organization of Medical Sciences (CIOMS), emphasising the importance of community engagement as a critical element of health-related research [12]. Consultation or engagement with communities has been considered as a tool for mitigating potential nascient ethical delemias within development or health projects. In the context of public health work within the global South, Adhikari et al. [13] states that community engagement is stressed as something to be deployed instrumentally, focusing on its ability to galvanise project delivery, with less emphasis on ethical good practice. Intrinsic and explicit constraints placed on projects in the global South by those that fund them, very often from a country in the global North, tend to focus on outputs or at best outcomes that largely ignore process and that which are intangible within the affected communities. Lack of effective engagement, particularly in low income countries where there are disparities in education, economy and power, can contribute to suspicion and study refusals [14].

In terms of drone use, central to aviation law is the safty of people and property on the ground and the safety of other airspace users, commonly enforced through the requirement for drone pilots to have a recognised qualification demonstrating competency and understanding of these laws [15]. Additonally, aviation authorities define zones where drone flights are resistricted (e,g, in the proximity of aerodrones where specifc permission must be obtained) or zones where flights are prohibited (e.g. government facilities and sensitive infrastructure). However, there is no legal or procedural requirement to engage the communities over which the drones fly. Services exist (mainly within more economically developed countries) that allow members of the general public to define regions of Drone No Fly Zones over their personal property (e.g. services such as www.noflydrones.co.uk) but there is no legal requirement for these areas to be respected in the same way as controlled or restricted airspace. Not only does this represents an omission ethically but unsolicited drone use could contribute to a loss of acceptance and support for wider public health initiatives. Despite this importance, currently there have been no publications for capturing this type of data in the context of drone use in malaria control.

This study presents a methodological framework to address these ethical considerations through the development of a community engagement framework. This framework draws on two factors, one a profile of the potentially affected communities as key stakeholders, secondly a participatory mapping of community attitudes and perceptions of the use and acceptability of drones. The concepts of community and engagement are interpreted differently across the domains of health promotion and health related research [16]. For the purposes of this paper, communities are geographical, and engagement relates to all of the direct and indirect interactions between them and the project.

The study takes place in Zanzibar, United Republic of Tanzania, preceding an LSM programme that uses drones for mapping mosquito breeding sites. In the context of this project and the proposed use of drones, ethical considerations were determined, drawing on an understanding on the social, cultural and historical context for the research and community perceptions of the proposed research tools, primarily drones.

The specific objectives of this work were to: i) Identify key stakeholder groups within Zanzibar communities. ii) Determine prior undertstanding and perceptions of drone use. iii) Establish levels of trust of drone use within communities and the drivers of trust, whether in support of drone use or negative feelings towards drone use. iv) Perceptions on who should providing permission for drone deployment.

## 2 Methods

### 2.1 Study location

Zanzibar is a semi-autonomous archipelago in the western Indian Ocean, ∼130 km off the coast of Tanzania. The Zanzibar Malarial Elimination Programme (ZAMEP) has made great strides in their battle against malaria, chiefly through widespread bednet use and targeted indoor residual spraying of insecticide [[17], [18], [19], [20]]. As ZAMEP make a final push towards malaria elimination they are looking to integrate interventions such as LSM into their programme. In partnership with Aberystwyth University (UK), ZAMEP are trialing the use of drone and smartphone technology for supporting LSM activities.

In January-Feburary 2021, participatory mapping of community attitudes and perceptions of the use and acceptability of drone use was carried out across three villages with community councils (Shehias): Bumnwisudi, Ndagaa and Mahonda, Unguja island, Zanzibar. As well as being in close geographic proximity, the three communities were defined as being linked by social ties, common perspectives and interests. These communities present a representative sample of rural conditions, with a mixture of agriculture ranging from large scale irrigation to more small scale rainfall-fed farming. In terms of formal institutions, there are seven schools, four primary and three secondary in, or within close proximity to the three communities.

### 2.2 Community engaged research

The study adopted a community focussed approach, that incorporated a mix of qualitative and quantitative methods, underpinned by CIOMS ethical guidelines [12]. Through the study, community based stakeholder groups were identified and characterised. Key stakeholders were those individuals, entities and organisations in the project affected communities, who would/could be affected daily by use of drones, those that may have an interest in the research and those who could influence whether or not drone activity proceeded in the area. Each stakeholder group was then categorized by their relative influence (i.e. how powerful their influence is) and importance (i.e. those stakeholders whose needs and interests coincide with the aim of the drone-related activity).

Community stakeholder engagement, as a planned process, presents an opportunity to provide input into research to improve its outcomes and goals [21,22]. Engagement occurs along a spectrum: from reaching out and informing, to consulting, involving, collaborating and shared decision making, also known as empowerment [23]. The degrees of stakeholder engagement can be viewed as a continuum of potential influence on a decision or action being considered from the initial distribution of information through to stakeholder empowerment.

### 2.3 Stakeholder analysis

Understanding who to involve and how – from provision of information through to delegation of decision making – requires an understanding of the different stakeholder groups, their characteristics, interests in a project, influence over a project and importance of the project to their living realities.

Identifying and assessing the influence and importance of the different stakeholder groups involves a technique known widely as Stakeholder Analysis [24]. Analysing stakeholder groups according to how much their interests coincide with a project (importance) and their ability to affect the success of a project or in other words how powerful they are (influence) are accepted parameters for mapping stakeholders. Such analysis or mapping enables understanding of what drives different stakeholder's involvement, their potential impact on the success of a project and hence how and when they should be enaged with across a project life and how much attention to give to the respective stakeholders [25].

Stakeholder analysis involved four steps: 1) Identifying the different stakeholder groups; 2) assessing the nature of their respective influence and importance; 3) constructing a matrix according to their level of influence and importance; and 4) preparing and enagement framework based on the matrix.

Six key stakeholder groups were identified (Table 1): Shehia (smallest government administrative unit) committees; school management committees; school teachers; young people; men's groups; women's groups. Once the stakeholder groups are identified and characterised, summarising their relative influence and importance, it is possible to determine what level of engagement they would require in relation to the drone activities.

**Table 1:** Summary of community stakeholder groups their relative interest, influence and importance.

Stakeholder Groups	Community	Interest	Influence	Importance
Shehia Committees (SC)	Bumbwisudi Mahonda, Ndagaa (Ghana)	Live locally, Community Governance Structures	High	High
School Management Committees (SMC)	Chuini Mawimbini, Kitope, Mahonda, Mfenesini, Uzini	Live locally, Community Institution Management	Medium	High
School Teachers (ST)	Chuini Mawimbini, Kitope, Mahonda, Mfenesini, Uzini	Work in the community institutions (schools)	Medium	Medium
Young People (YP)	Bumbwisudi, Ndagaa, Mahonda	Live in the community	Low	Medium
Mens Groups (MG)	Bumbwisudi, Ndagaa, Mahonda	Live locally and work in community farming groups	Low	High
Womens Groups (WG)	Bumbwisudi, Ndagaa, Mahonda	Live locally and participate in community women's groups	Low	High

Once analysed and considered, each stakeholder group can be categorized according to their relating importance/influence, guided be the matrix in Table 2. Shehia Committees (SC), as local government representatives, are both influential and important and should be involved in decision making regarding how and where the drone activity is implemented. School Management Committees (SMC) are influential in the community but not very important to the drone activity. They should be informed and consulted with so they can feed into decision making. The community members, farming men's groups (MG) and women's groups (WG) and young people (YP), have very little influence but are very important to the drone programme and should be kept informed and consulted with so their views and opinions are considered in the research planning. School Teachers (ST), are not very influential or important to the drone activity but should be kept informed about what is planned, where and when.

**Table 2:**
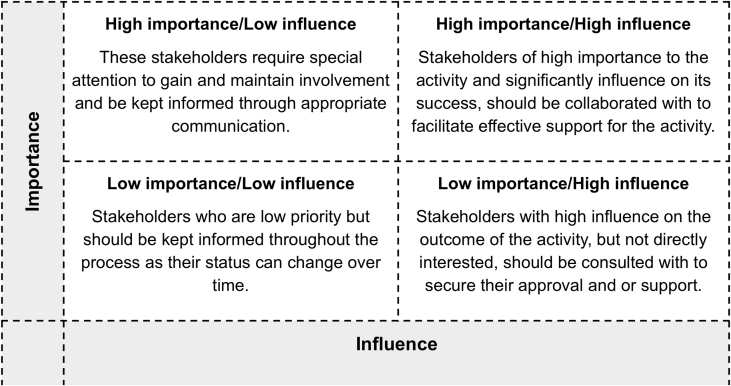
Stakeholder analysis matrix of importance and influence.

Following the Stakeholder Group analysis, an engagement framework was prepared based on the World Bank Participation Continuum [26]:

• **Informing**: one-way communication flow in which stakeholders are passive information receivers.
• **Consulting:** one-way, although there is an opportunity for stakeholder feedback to be received.
• **Involvement:** requires two-way interaction, entailing providing feedback on stakeholder contribution.
• **Collaboration:** involves developing stakeholder partnerships within decision making processes.
• **Empowerment**: the delegation of final decision-making (on identified issues) to stakeholders.

Stakeholder participation and impact on the process increases along the continuum, summarised and the project linked to the stakeholder analysis of influence and importance so that the community stakeholders in the study area with: low influence and importance were kept informed; low influence and high importance were informed and consulted; high influence and low importance were informed and consulted; high influence and importance were informed consulted and involved in decision making.

### 2.4 Data collection

To understand what type of information should be shared, what issues the community should be consulted on and involved in, it was important to understand existing attitudes towards and perceptions of drones. A mixed-methods research approach [27] was adopted combining qualitative and quantitative data collection methods using questionnaires, semi structured interviews and focus groups. Questionaires were prepared using ArcGIS Survey 123 (version 3.13) software that enabled geotagging of all of the data collected. A set of survey questions were prepared with predetermined answers using likert scales to capture the three communities perceptions of drones. Focus group and semi structured interview guides were also produced that followed a similar narrative to the questionaires, without predetermined answers.

The survey tools were presented to the Zanzibar Ethics Committee for review and approval. Their comments were addressed and the three study tools were deployed by a data collection team comprised of two men and two women from Sazani Trust, Zanzibar. Individual identities of study participants were kept confidential, only data related to gender, age and location was made explicit. Questionaires were undertaken with a purposive sample of stakeholders from each of the identified stakeholder groups in each of the communities. Focus groups were undertaken with small representative groups of the respective stakeholders and semi structured interviews were carried out with key informants from each of the stakeholder groups. Cultural sensitivity linked to gender was applied to the research methodology, with males and females being surveyed and or engaged with separately to foster interactions that permitted expressions of gendered identities, roles, and experiences.

### 2.5 Cost data

As a potentially important component for the operational deployment of drones, we sought to collate and present the costs associated with the community drones perception study. Health system cost data was collected retrospectively through record review. The quantity, timings and unit costs of each member of staff have been collated to calculate total staff costs. Consumables and transport costs such as fuel, stationery, vehicle rental, etc. have been collected through a mix of direct observation and record review.

We also included costs related to lost productivity for the community members who participated in interviews, using mean interview duration and World Bank's GDP per capita estimates for Tanzania. Sheha committee members indirect costs have been calculated using daily allowances from ZAMEP. As interviews took place in the community, it was assumed that community members were not required to travel, so no travel costs have been included for this. Similarly, it was assumed that their lost time did not exceed the interview's duration.

Costs collected in the local currency have been coverted to US$ using the exchange rate reported by OANDA (www.oanda.com) at the time of the analysis. No inflation rate has been used as all costs reported are 2021 prices.

## 3 Results

### 3.1 Study participants

In total we had 778 participants in the study spanning the range of community and stakeholder groups with 19 groups across the three communities (Table 3). Overall, 60% of study participants were from Womens Groups, representing the rural culture of women in Zanzibar engaging in collective activities. Young People also represented a dominant stakeholder group accounting for 17% of total participants. Other groups (School Committees, School Teachers, School Management Committees, Mens Groups) represented between 2 and 8% of total participants.

**Table 3:** Breakdown of study participants per community and stakeholder group.

	School Commmittees	School Management Committees	School Teachers	Mens Groups	Womens Groups	Young People	TOTALS
Ndagaa	20	5	9	20	246	30	**330**
Bumbwisudi	26	4	4	5	40	11	**90**
Mahonda	8	12	24	41	183	90	**358**
**TOTALS**	**54**	**21**	**37**	**66**	**469**	**134**	**778**

### 3.2 Responses

**Fig. 1:**
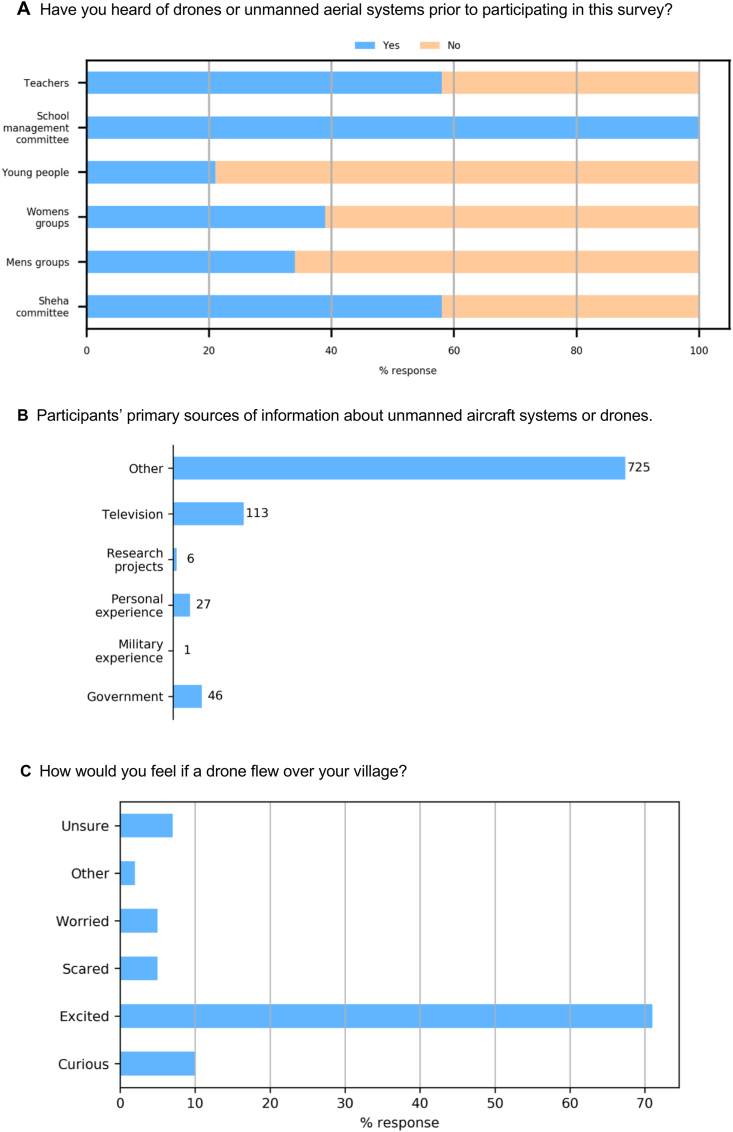
Breakdown of responses to the questions A: Have you heard of drones or unmanned aerial systems prior to participating in this survey? B: Participants' primary sources of information about unmanned aircraft systems or drones. C: How would you feel if a drone flew over your village?

*Have you heard of drones or unmanned aerial systems prior to participating in this survey?* There was very little difference in response to this question between the communities, with 59% responding “no” and 41% responding “yes”. On showing the participants a picture of a drone, their responses did not change significantly, suggesting that awareness of drones could be linked to education and community member exposure to different localities. Awareness could also be linked to internet access - In 2018, over 95% of Tanzania's 23 million internet users accessed the internet via mobile phone. Stakeholder groups where “yes” responses exceeded 50% (teachers, school management committees and Shehia committees) are all associated with a higher level of education and/or experience (Fig. 1A).

*What are [participants’] primary sources of information about unmanned aircraft systems or drones?* Most participants across the stakeholder groups and communities selected other from a detailed list of options (Fig. 1B). The primary source of information was political rallies and meetings, attributed to the recent national elections in October 2020 and the use of drones by state media and others to capture media footage of these meetings. Television was a significant source of information in Mahonda, but not in the other two communities, attributed to availability of electricity and TV reception. Given the relatively small number of responses (n = 27) that mention personal experiences suggests that show-and-tell and/or demonstration flights would be a potentially valuable mode of increasing community knowledge and awareness of drone technology.

Respondents were asked for true, false or unsure responses to the following statement: *Special approval from the Government is required to legally operate Unmanned Aircraft Systems in Zanzibar.* The Zanzibar Archipelago has a robust state governance structures, with state representation starting at the community level (Shehia Committees) and no parallel traditional/tribal governance structure. This, combined with a legacy of state socialism, means that governmental permission and approval is regarded as essential (96% responding “true”). The majority of respondents (76%) answered “true” when asked for true/false/unsure responses to the statement *Most unmanned aircraft systems currently in use are capable of operating completely autonomously without any human controller***.** Although it is true that most commercially available drones can be operated autonomously a human controller is always necessary. This perhaps relays a lack of knowledge within the rural communities, as to the nature of drones and how they are controlled: Something that could be easily remedied through community demonstrations prior to the drones being deployed.

When asked *How would you feel if a drone flew over your village?* The responses in each of the communities were positive with 71% suggesting that they would feel excited and a further 10% suggesting that they would be curious (Fig. 1C). Interestingly, negative responses, all from communities in Ndagaa and Bumbwisudi, correlate with exposure participants have had to drones, reinforcing the need for community-based demonstrations prior to being deployed in the field.

**Fig. 2:**
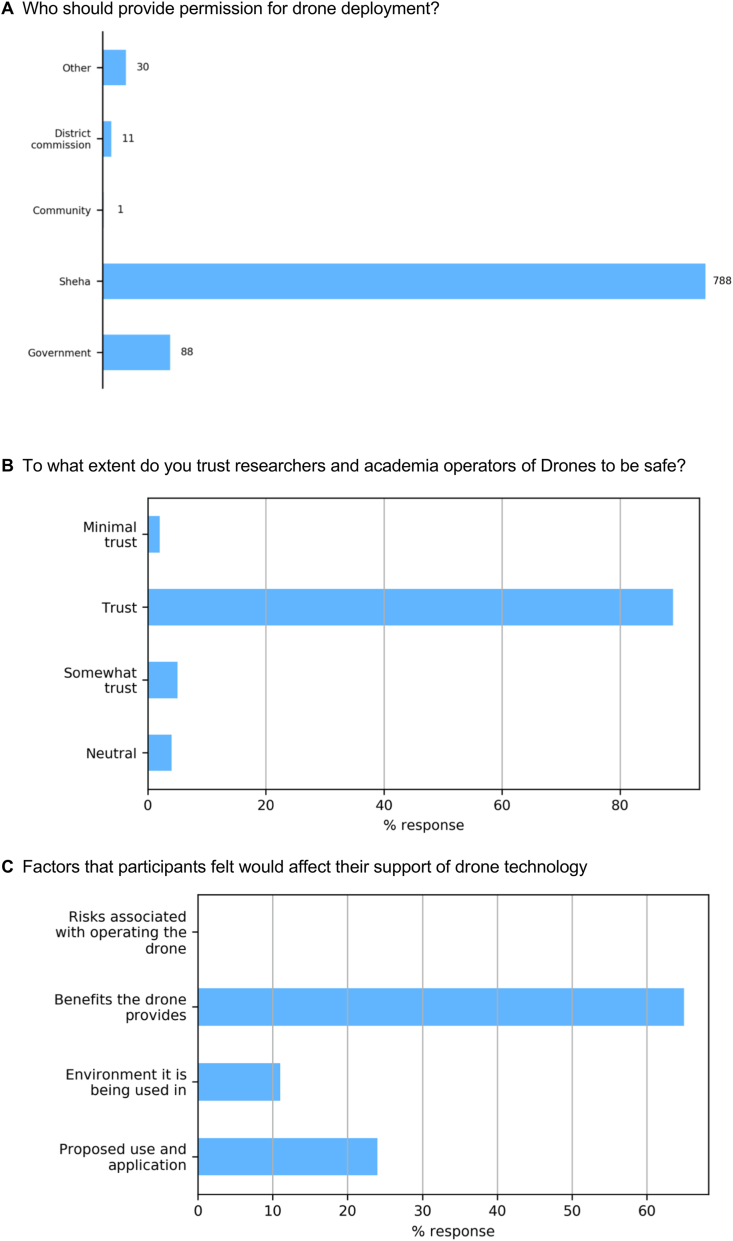
Breakdown of responses to the questions A: Who should provide permission for drone deployment? B: To what extent do you trust researchers and academia operators of Drones to be safe? C: Factors that participants felt would affect their support of drone technology.

Stakeholder groups were asked *whether they thought they should be notified before a drone survey is carried out, and who should provide permission deemed to be required*. Overwhelmingly (91% answering “yes”), people want to be informed before drones are deployed, with just four women's groups (from all three communities), not needing to be notified beforehand. Most respondants felt that permission should come from the Sheha (head of the Shehia administrative area) (Fig. 2A) demonstrating their importance in community-level decisions, with teachers and some students also requiring permission at a Ministerial level from the Government.

Respondents, within focus group discussions, were asked questions regarding their perceived safety of drone operations and benefits that drones could bring to society. A vast majority of participants felt that drones were safe for people and buildings (83%: very safe, 12%: quite safe; 5% not safe), and 94% of respondents that felt that drones were beneficial to society (6% were unsure) with no one of the opinion that drones offered no benefit. This overall positive outlook on drones is supported by a good level of trust (89%: Fig. 2B) within communities when asked about the extent at which respondents trust drone operators to be safe, mirroring findings made in a previous study in Dar es Salaam, Tanania, with (78%) of the witnesses to drone demonstrations having no concerns about the use of UAVs in their communities [28].

Importantly, despite a high level of trust in drones and perceived benefits they offer, the support for drone use was not unconditional: according to respondents from across the stakeholder groups and the three communities, acceptance was linked mainly to the perceived benefits (65%: Fig. 2C), but also the environment they are being used in, and the purpose of its application.

### 3.3 Cost data

#### 3.3.1 Health system costs

Staff costs are the most important cost driver, with data collection costing up to US$11,167. Given the large number of study participants, this required 10 days of intensive work and four staff members. Other staff costs related to study preparation activities such as conceptualising the research framework and report preparation were less significant because these were largely desk-based activities. Table 4 gives details of the health system costs incurred. It shows that overall, collecting the data imposed substantial costs, while the costs of implementing the study results represent just 11% of the total health system costs.

**Table 4:** Health system costs associated with the drone perceptions study and indirect costs associated with study participants.

Health system costs				Study participant costs		
Cost category	Activity	Cost US$	% of Total	Cost category	Activity	Cost US$
**Sazani Staff**	Data collection	11,167	46%	**Shehia**	Individual interviews and focus group discussions	22
	Data analysis and report preparation	6980	29%			
	Admin and support	1565	6%	**Community member**	Individual interviews and focus group discussions	241
**Transport**	Vehicle fuel and driver	430	2%			
**Equipment**	Tablets	558	2%			
	Research software license	419	2%			
**Research**	Ethics	500	2%			
**Study implementation**	Posters design	2792	11%			
**Total**		**24,411**		**Total**		**263**

#### 3.3.2 Study participants costs

Study participants costs (Table 4) have been calculated using the 15 min mean interview duration that was lost to other activities such as work. These indirect costs were calculated separately for all the 54 Shehia committee members and the 724 other community members. This is because data on the daily allowances were collected for Shehias only and this was equivalent to US$35.4 per day. As stated in the Methods section, for the other community members, World Bank estimates on GDP per capita have been used. This was equivalent to US$10.7 per day. These costs represent only 1% from the total costs, however, the absolute value as a whole is not important, but the impact it has on the work-related activities.

A costing analysis of running a drone-based mapping of malarial mosquito breeding sites for targeting larval source management interventions is currently underway, but we estimate that the drone perception study would represent approximately 25–35% of total economic costs.

## 4 Discussion

For the communities examined in Zanzibar there was a high level of acceptance of drones and their usage for research related activities. This represents a refreshing finding given the broader-scale negative connotations associated with drones due to their use in military operations [29] or perceptions of being risky technology that might interfere with privacy, particularly in relation to their use commercially (e.g. use in delivery of products) or by hobbyists [9,10,30].

The perceptions mapped in this study relate to a research project using drone technology to support malaria control initiatives. As such, opinions and perceptions of drones are framed within the context of a clear and relatable benefits to the communities surveyed. The positive support of drones within Zanzibari communities aligns with findings made in other studies that identify a high level of support for the use of drones in scientific research [10,28]. However, this support may be fragile: the use of drones in less favourable applications (e.g. use in media, deliveries, hobbyists) could easily undo the support for drone applications with a clearer route to public good, such as public health and safety, e.g. for use by fire services [31]. This may be confounded where drone use is unsolicited, without engagement with local communities.

Despite the general support and acceptance of drone use in Zanzibar the exposure to drones and prior knowledge is low, with over half of respondents not having heard of drones before, compared to other studies based in the US with a near universal awareness of drones, particularly through mainstream news media [32]. In this study, the communities, with the exception of Mahonda were all very rural with out access to internet and predominantly without smart phones. In this respect, acceptance in Zanzibar may actually be a function of community trust in their governance and leadership: if drone operations have been permitted, then it must be beneficial. As described by one participant: “I trust them because the government gives them permission”.

Given the low rates of exposure and awareness it is clear that a community engagement plan needs to involve a non-technical, introduction and demonstration of drone technology and what benefits they can offer (in the context of public health). Equally, given the levels of influence and trust in local governance, it is important that permissions and consent are sought from Shehia Committees (the smallest unit of governance in the Republic of Tanzania), again, so that high levels of support for drone use is not undone.

Valid informed consent is a critical element of ethical health-related research but often in cluster-based studies, this consent is sought from government representiatives rather than community members. As the study has shown, initial engagement through the drone perceptions study presented the first step in providing community stakeholders with accurate and adequate information about the study. With perceptions and experiences varying from one region to another, it is important that this kind of study is implemented before drone operations are deployed Understanding what is proposed and being involved in a continuous dialogue through appropriate community engagement will be the next step [33]. Facilitating community engagement has been shown to improve the validity of consent, by enhancing understanding of what is expected and why [34]. This in turn contributes to gaining both formal and informal permissions, approvals and legitimacy for a planned study [21].

In Zanzibar, a large proportion of respondants felt that permission for drone operations should be sought from the communities via Shehia Committees. As such, community engagement should be considered a pre-requsite to all programmes where drones are employed. Additionally, there was a strong desire to be kept informed about these activities. This was deemed to be important not only at the Shehia Committee level but also with rural communities and school management committees informed through regular meetings. There are costs related to doing this but in addition to the possible benefits regarding future operational activity, ethically it moves any future engagement towards a form of collaboration with the local population. In doing so, malaria control programmes reliant on drone use can be sustained for the foreseeable future, secure in the knowledge that they have the consent and support from local communities. Designing future research alongside host communities as collaborators and inheritors of technological approaches would be the ideal scenario.

### 4.1 Engagement plan

An engagement plan was developed to plan and deliver an appropriate community engagement process to keep the right people engaged in drone-related activity with the right amount of detail – a critical component to making stakeholders feel valued, involved, heard, and appreciated. Specifically, it was important to map stakeholder groups with the purpose of the engagement activity, the methods and frequency of engagement, those responsible for delivering this engagement and a clear plan for reviewing each of these components.

Methods of engagement were simple: demonstration/information events, project information sheets and meetings are recommended with key stakeholder groups including Shehia Committees, Community groups (Men's groups, Women's groups, youth groups) and school management committees (Table 5). These are to be delivered prior to drone deployment but also, in the case of project information sheets and meetings should be ongoing (every two-three months), informing stakeholder groups of progress and updates, but also re-mapping perception to record and react to any changes in trust or acceptance. Costs related to this ongoing engagement were not collected in this study. An evaluation of the engagement plan was not carried out in this study but represents an important direction for future studies to determine the effectiveness of these types of tools.

**Table 5:** Overview of drone-activity engagement plan for communities in Zanzibar.

Key stakeholders	Purpose of engagement	Engagement method	Frequency	Responsibility	Review
Shehia Committees	To build on current trust and secure and maintain formal consent for drone usage	Demonstration/Information events	Prior to drone deployment	Aberystwyth Uni, ZAMEP	After each event to see how it could be improved
		Project information sheets	Prior to drone deployment and ongoing	Aberystwyth Uni, ZAMEP	Update every six months
		Meetings	Prior to deployment then every 2–3 months	ZAMEP, community consultants	Ongoing
Community members (men, women, youth)	To maintain trust and informal consent for the drone usage	Demonstration/Information events	Prior to drone deployment	Aberystwyth Uni, ZAMEP	After each event
		Project information sheets,	Prior to drone deployment and ongoing	Aberystwyth Uni, ZAMEP	Update every six months
School Management Committees					
		Community meetings	Prior to deployment and the twice a year with updates	ZAMEP, community consultants	After each meeting
Teachers	To keep informed	Project information sheets	Prior to drone deployment and ongoing	Aberystwyth Uni, ZAMEP	Update every six months

### 4.2 Costs

Some cost components, such as ethics approval costs might be relevant for future studies, but not for studies conducted by ZAMEP. Health system factors, such as wages and prices can influence costs. For example, the use of ZAMEP staff may drive the staff costs down by 67%. However, this might require extensive training (in qualitative research) and supervision which have not been taken into account in this additional costing calculation.

Total time lost by community members due to their involvement in the project might not be equivalent to the interview duration, but these timing data have not been collected as part of this study. As a result, community members indirect costs could have been underestimated. However, we are confident that this would have not meaningfully changed our findings. Although not used in this study, time lost compensations for community members are common practice in qualitative research, and might need to be considered in future studies.

## 5 Conclusions

Permissions for flying drones are necessary in most countries across the World. Although definitions and terminology can be ambiguous at times most civil aviation authorities will expect specific permission related to commercial or governmental drone activities to ensure drone operators a suitably qualified, have insurance cover and have procedures to maximise flight safety and accountability. But currently, for most regions of the World, permission and consent is not required from communities where drone activity is planned. This may be particularly important given the potential disparities in power between the global North and South, particularly using drone technology and their association with an invasion of privacy. Indeed, for countries like the UK the use of drones for collecting images may fall under specific data protection legislation (Such as the Data Protection Act 2018) which must be considered for ethical approval but in the United Republic of Tanzania a specific data protection bill is currently in draft but not yet law.

This study, focusing on communities in Zanzibar in the context of a malaria control intervention supported by drone technology, represents a methodological framework for mapping community attitudes and perceptions of the use and acceptability of drones. We argue that from an ethical perspective, this kind of study should be a pre-requisite for any drone activity taking place within, or near communities, regardless of application. In sectors like public health, there is a growing assertion that drones can add benefit, perhaps even step change improvements to the way we deliver important public health services and programmes. Yet if these activities are to be successful and sustainable we need to apply appropriate and effective community engagement strategies. Similarly if global health interventions want to avoid being labelled as emanating from the global North as paternalistic or experimental science projects then engagement must recognise the power imbalance and attempt to address it. Failure to do so is not only unethical but leaves important interventions vulnerable in an era of social media.

The key components in this work included the engagement process and implementation of a resulting engagement plan costing US$21,619 and UA$2179 respectively. We recommend that this is a valuable investment in terms of the long term sustainability of the drone programme and, importantly, reduce the risk of community disengagement/distrust which would be damaging in both tangible (project outcomes) and intangible (once broken relations are hard to repair, plus part of wider decolonization agenda) ways.

This study revealed widespread trust and support for drone activities for use in malaria control research. But crucially, this support is not unconditional: all stakeholder groups need to be informed prior to drone deployment and consent given; demonstration or information need to be delivered; regular engagement activities need to be conducted, such as meetings and information sheets, to update stakeholders. Ultimately, although trust exists within the Zanzibarian communities studied, this trust can be easily undone, but suitable engagement plan can provide a simple but effective means of building and maintaining trust and acceptability.

## Funding sources

This study was funded by the 10.13039/100000865Bill and Melinda Gates Foundation Innovation Fund INV-010583 through a grant managed by the Innovative Vector Control Consortium (IVCC).

## Ethics statement

Steps were taken to ensure confidentiality for all those who participated in the research and that any data stored was anonymised and protected under Sazani Associate's data protection policy. All the team members (all based in Zanzibar) have current CRB checks and Sazani have just had their safeguarding systems reviewed very recently by UK Department for International Development. Informed consent for participation in the research was gained verbally, this deemed to be appropriate given the relatively low rates of literacy. Our study was approved by the Zanzibar Health Research Institute reference ZAHREC/03/AMEND/OCT/2020/07 on 29^th^ September 2020.

## Author contributions

**Andy Hardy**: Conceptualization, Methodology, Formal analysis, Investigation, Writing – original draft, Project administration, Funding acquisition. **Mark Proctor**: Conceptualization, Methodology, Investigation, Writing – original draft, Supervision, Project administration. **Cathryn MacCallum**: Conceptualization, Methodology, Investigation, Writing – original draft, Supervision, Project administration. **Josh Shawe**: Methodology, Formal analysis, Investigation. **Safia Abdalla**: Methodology, Investigation, Resources, Data curation. **Rajab Ali**: Resources, Data curation. **Salha Abdalla**: Resources, Data curation. **Gregory Oakes**: Methodology, Investigation, writing –; reviewing and editing. **Laura Rosu**: Methodology, Formal analysis, Investigation, Data curation, writing –; reviewing and editing. **Eve Worrall**: Methodology, Formal analysis, Investigation, Writing – original draft, Funding acquisition.

## Declaration of competing interest

The authors have no competing interests to declare.
